# Tracking the pandemic through molecular and sequencing tools: a story of SARS- CoV-2 over five years, lessons learned, and further directions

**DOI:** 10.1186/s12879-025-12200-x

**Published:** 2025-11-25

**Authors:** Saeed Khan, Hafsa Faruqui, Maria Zahid, Sharjeel Chaudhry, Zaira Rehman, Hamza Noor, Manaal Naushad

**Affiliations:** 1 Provincial Public Health Laboratory (PPHL) Sindh, Dow University of Health Sciences, Karachi, Pakistan; 2 Department of Molecular Pathology, Dow Diagnostic Research & Reference Laboratory, Dow University of Health Sciences, Karachi, Pakistan; 3 Indus Hospital & Health Network, Karachi, Pakistan; 4 Dow International Medical College, Dow University of Health Sciences, Karachi, Pakistan

**Keywords:** SARS-CoV-2, COVID-19, Genomic epidemiology, Surveillance, Pakistan, Alpha, Delta, Omicron, In-house PCR, Whole genome sequencing

## Abstract

The COVID-19 pandemic posed unprecedented challenges to public health surveillance frameworks. In response, the Provincial Public Health Laboratory (PPHL), Sindh, contributed to early detection, large-scale diagnostic testing, and genomic surveillance from February 2020 to June 2025. This study reviews five years of PPHL’s role in COVID-19 diagnosis, variant tracking, and public health response in Sindh. Diagnostic data included positive cases, while genomic epidemiology was assessed using commercial RT-PCR kits and in-house S-gene dropout assays as proxy tools for variant detection, supplemented by whole genome sequencing (WGS). A total of 194,415 specimens were tested, with five major peaks observed: wild-type (33.3%), Alpha (26.9%), Delta (22.0%), and Omicron (23.1%). WGS was performed on 124 selected samples, which identified Delta (AY.127, B.1.617.2), Omicron (BA.1, BA.5.2, XBB.1.9.1), and, more recently, JN.1, BA.2.86.1, and XEC lineages in May 2025. Reports of NB.1.8.1 from neighboring countries were noted, but this lineage was not detected in Karachi samples. While study limitations like low portion of sequenced samples and potential bias toward low Ct specimens exist, these findings still highlight the value of sustained laboratory-based surveillance in documenting SARS-CoV-2 evolution and supporting public health decision-making in Sindh.

**Supplementary Information:**

The online version contains supplementary material available at 10.1186/s12879-025-12200-x.

## Clinical trial

Not applicable.

**Supplementary Information:**

The online version contains supplementary material available at 10.1186/s12879-025-12200-x.

## Introduction

As of the most recent data reported by the World Health Organisation (WHO), a total of 778,186,599 COVID-19 cases and 7,096,935 deaths have been cumulatively documented worldwide [1]. The global emergence of the novel coronavirus SARS-CoV-2 in late 2019 led to an unprecedented public health emergency.

Pakistan experienced major waves of the pandemic between 2020 and early 2022, in the following periods: March to July 2020, October 2020 to January 2021, March to May 2021, July to the end of September 2021, and December 2021 to February 2022. As of June 2025, Pakistan had reported approximately 1.58 million confirmed cases of COVID-19, resulting in about 31,000 related deaths. Of the COVID-19 cases in Pakistan, 36% originated from Sindh, with 40% of them occurring in Karachi. The case fatality rate (CFR) has been 2% with few regional variations [2]. While there was no country-wide wave of large magnitude during 2024, localised surges in cases were still seen in Pakistan, primarily in urban centres such as Karachi. These increases in cases coincided with the emergence of global Omicron subvariants and recombinant lineages, namely JN.1 [3]. In early 2020, as the cases of COVID-19 surged worldwide, the healthcare systems were confronted with the immediate necessity to rapidly expand diagnostic capacity, initiate surveillance, and counter emerging variants [4].

Within the initial months of the pandemic, SARS-CoV‐2 began accumulating mutations in its genome. Among the most notable mutations were those occurring in the spike glycoprotein, due to its implication and mediation for host cell entry using the angiotensin-converting enzyme 2 (ACE2) receptors. Variants with significant spike gene mutations were designated as Variants of Concern (VOCs) by the World Health Organisation (WHO), including lineages such as Alpha (B.1.1.7), Beta (B.1.351), Gamma (P.1), Delta (B.1.617.2), and Omicron (B.1.1.529). VOCs exhibit gene mutations linked to increased transmissibility, increased severity of disease, reduced effect of vaccination or medical treatment [5]. The Omicron strain that emerged in the latter part of 2021 harboured over 50 mutations across the genome, resulting in reduced vaccine effectiveness with a greater chance for breakthrough infection. Omicron was also more transmissible than other strains, with a greater chance of reinfections. Moreover, it was associated with lower in‐hospital mortality rates.

Provincial Public Health Laboratory (PPHL) in Sindh played an important role in the provincial and national COVID-19 response by spearheading the development of diagnostic protocols and leading efforts in variant detection throughout multiple waves of the pandemic [6]. Anticipating the inevitability of cross-border transmission that is unavoidable, the PPHL Sindh was among the first regional laboratories to develop an in-house real-time RT-PCR test for SARS-CoV-2 detection. Over these five years, the laboratory documented evolving positivity rates after the emergence of various VOCs and their sublineages.

To enhance variant detection capabilities, we designed an in-house dropout PCR assay that targets the 69/70 deletion in the spike protein to facilitate rapid screening of Alpha and Omicron cases, both VOCs that carry this significant mutation. From 2021 to 2025, a subset of positive samples was sequenced at different time intervals for continuous genomic surveillance of circulating SARS-CoV-2 variants and strains. A local outbreak in May 2025 also showed the presence of recombinant lineages like XEC in the population, which signifies continuous viral evolution and highlights the necessity of continuous genomic surveillance. This article reflects a five-year retrospective overview of PPHL’s testing, diagnostic innovation, and genomic monitoring throughout Pakistan’s COVID-19 response.

## Materials and methods

### Study design and setting

This retrospective study was conducted at the PPHL, Sindh, Dow University of Health Sciences (DUHS), from February 2020 to June 2025. It spans the beginning of the COVID-19 pandemic, all of the subsequent major waves, and minor localised outbreaks that occurred in the 5 years.

### Sample collection and processing

Nasopharyngeal swabs were collected from referred suspect COVID-19 patients from various health facilities, quarantine camps, and community screening drives in the Sindh province. The samples were transported to the PPHL in viral transport medium (VTM) under the cold chain by biosafety and sample handling guidelines issued by the World Health Organisation (WHO) and NIH Pakistan. A total number of 194,415 tests were performed between February 2020 and June 2025.

### RNA extraction

Viral RNA was extracted from the swab samples using Qiagen QIAamp Viral RNA Mini Kit (Qiagen, Germany) [7]. The procedure followed the manufacturer’s instructions, eluting RNA in 60 µL of elution buffer. Sample integrity and RNA yield were monitored through spectrophotometry and internal control amplification.

### RT-PCR assays for SARS-CoV-2 detection

When cases of COVID-19 began spreading globally, the PPHL predicted the virus to soon cross borders into Pakistan as well. To prepare for its testing and detection, firstly, an in-house RT-PCR diagnostic assay was prepared using previously published primers that targeted ORF1ab and Nucleocapsid [8]. A Ct value ≤ 38 was positive based on national guidelines. Quality control was ensured by the inclusion of positive and negative controls with every run. Later, commercial kits like the Cobas SARS-CoV-2 real-time RT-PCR kits were used for large-scale testing and diagnosis of samples on the Cobas 6800 system (Roche Diagnostics, USA).

### Variant detection: dropout PCR and RT-PCR kits

To identify Variants of Concern (VOCs), specifically Alpha and Omicron lineages, an in-house dropout PCR for the Δ69/70 deletion in the spike (S) gene was developed in late 2020, upon the arrival of the Alpha variant [9]. To exploit this unique genomic signature for rapid screening, we designed a set of PCR primers flanking the 69–70 region. Crucially, a variant-specific primer was incorporated that would only bind to and amplify the intact (non-deleted) 69–70 region. In samples containing the deletion, this primer would fail to anneal, resulting in a “dropout” of the expected amplicon, thereby providing an indirect yet effective method for detecting the deletion. Following thermocycling, PCR products were analysed using 2% agarose gel electrophoresis. The presence of two bands (158 bp and 280 bp) indicated a wild-type strain, signifying the absence of the 69/70 deletion. Conversely, a single band of 280 bp specifically signified the deletion, thereby identifying the presence of either the Alpha or Omicron variant. Confirmation was done by taking a subset of dropout-positive samples (*n* = 15) and subjecting them to Sanger sequencing, which was then interpreted using MEGA7 and Chromas software. Soon after, RT-PCR kits had been developed for the rapid and cost-effective screening of VOCs, and so the SNPsig EscapePLEX SARS-CoV-2 (Primer Design, UK) was used for continuous variant surveillance.

### Whole genome sequencing (WGS)

In efforts to strengthen genomic surveillance in the province, PPHL Sindh began NGS analysis of SARS-CoV-2 positive samples in 2021. A fraction of low Ct values (< 30) PCR-positive and epidemiologically significant samples was selected for sequencing. Two main platforms were used for NGS—IonTorrent (ThermoFisher, USA) and Oxford Nanopore Technologies (ONT, UK). Library preparation was done using recommended protocols for both platforms, namely the Ion AmpliSeq™ SARS‑CoV‑2 Research Panel (ThermoFisher, USA) and the NEBNext^®^ ARTIC SARS-CoV-2 Companion Kit (New England Biolabs, USA), respectively. Genome assemblies were checked for lineage classification by the Pangolin and Nextclade tools. Read depth, genome coverage, and alignment accuracy were used as quality control parameters. A total of 124 SARS-CoV-2 sequences from PPHL Sindh have been uploaded to GISAID.

### Data management and analysis

Demographic and clinical metadata were collected on requisition forms and laboratory reports. RT-PCR and NGS data were collated in Microsoft Excel and analysed using SPSS v25.0 for descriptive statistics [10]. Age, sex, Ct values, test positivity, and variant distribution over time were some of the key variables.

Ethical approval was obtained from the Dow University of Health Sciences Institutional Review Board [Ref: IRB-1649/DUHS/Approval/2020/195], and all processes were performed under approved public health surveillance protocols. Confidentiality of the patients was maintained during the study.

### Phylogenetic analysis

After removing low-coverage sequences, we performed whole-genome and phylogenetic analysis of 50 SARS-CoV-2 sequences obtained from this study, in addition to 46 genomes available from different countries on GISAID (Accession IDs in Supplementary Table 2). Multiple sequence alignment was performed using the MAFFT tool on the Galaxy platform. The aligned sequences were then subjected to maximum likelihood phylogenetic inference using IQ-TREE with automatic model selection. The resulting tree was visualised and interpreted using FigTree.

## Results

**Fig. 1:**
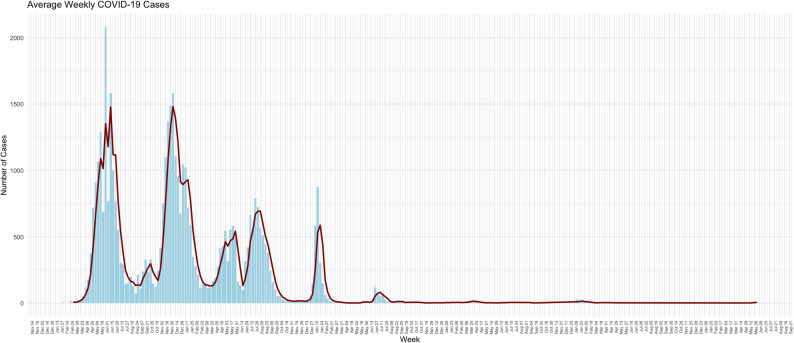
Shows average weekly positive cases during the five years of COVID-19

A total number of 194,415 tests were performed between February 2020 and June 2025. From these tests, 43,430 were positive cases with an overall positivity rate of 22.3%. From the positive cases, 62.6% (*n* = 27,190) were males 37.4% (*n* = 16,236) were females and < 1% (*n* = 4) were transgenders. The mean age of positive cases was 40.6 years, with a minimum age of 1 and a maximum age of 103 years observed. Figure 1 shows average weekly-positive cases during the five years of COVID-19, and as seen in the figure, the five distinct peaks each correlate with a different wave of the pandemic. Throughout the COVID-19 pandemic, five major waves of infection were documented worldwide, each characterised by distinct epidemiological patterns and driven by the emergence of new SARS-CoV-2 variants. Pakistan mirrored this global trajectory, experiencing five comparable waves of varying intensity and duration. Notably, each wave in Pakistan coincided with the emergence and widespread transmission of a specific variant of concern (VOC), or in some cases, with concurrent circulation of multiple variants. These variants (Alpha (B.1.1.7), Beta (B.1.351), Gamma (P.1), Delta (B.1.617.2), and Omicron (B.1.1.529 and its sublineages)) were associated with increased transmissibility, partial immune escape, or altered disease severity, contributing to the successive surges in case numbers. As depicted, the timing of these waves aligns closely with the detection and subsequent dominance of each VOC within the population.

### Initial testing and first wave (April–August 2020)

During the initial phase of the pandemic in Pakistan, specifically from April to August 2020, the PPHL conducted 44,984 tests, with 13,466 samples yielding positive results, indicating a 29.9% positivity rate. This period marked Pakistan’s first significant wave, characterised by the spread of the wild-type SARS-CoV-2 strain. The initial wild-type SARS-CoV-2 strain, were dominated during this time, and no variant tracking strategy existed [11]. PPHL rapidly scaled up molecular testing and played an important role in tracking the early surge.

### Alpha variant wave (October 2020–February 2021)

**Fig. 2:**
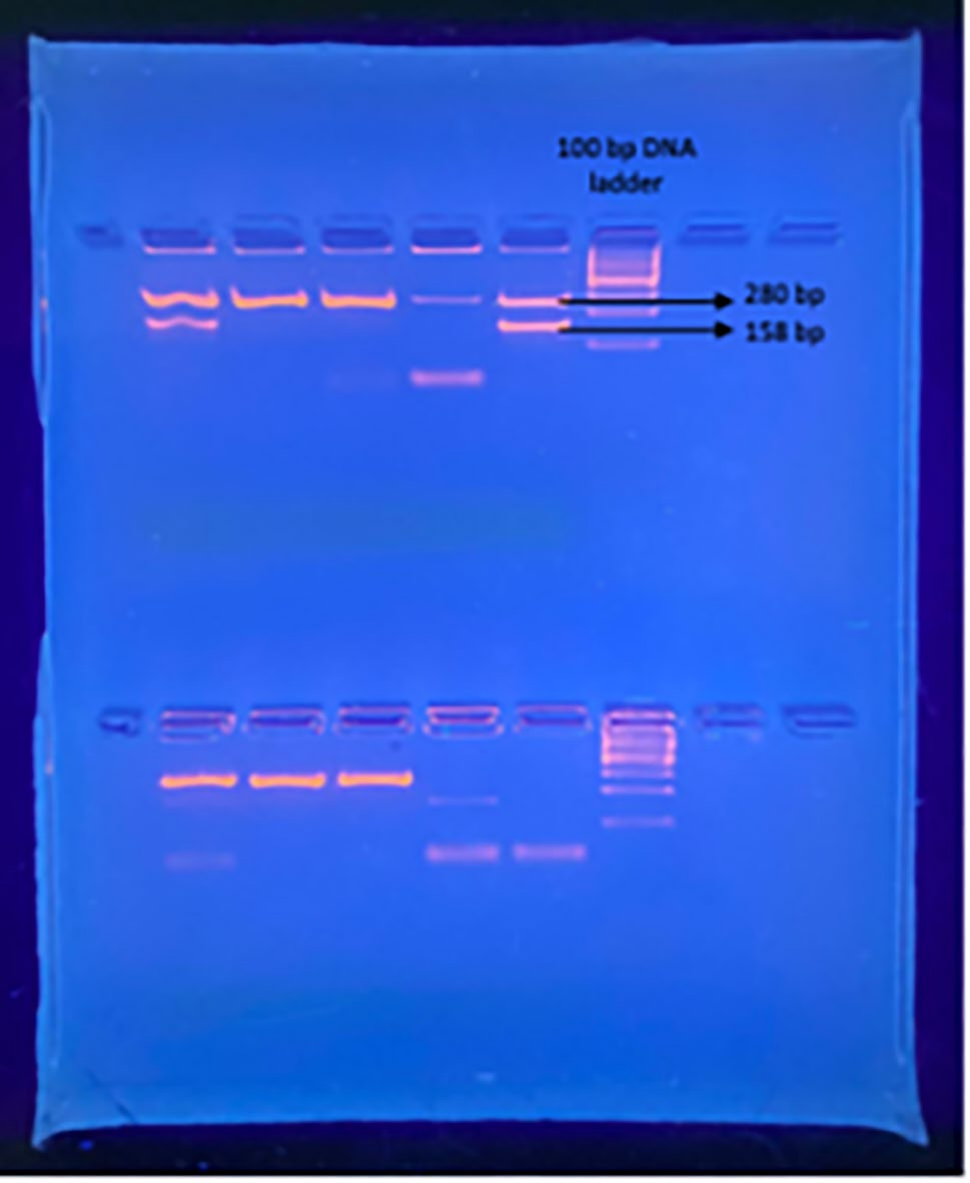
2% agarose gel electrophoresis showing bands of 280 bp and 158 bp for wildtype strains and a dropout of only 280 bp representing the 69/70 deletion. DNA marker ladder of 100 bp was run for reference size. Full-length gel is presented in Supplementary Fig. 1A

The second wave was followed by the detection of the Alpha variant (B.1.1.7) in Pakistan. The arrival of the Alpha variant (B.1.1.7) signalled a new chapter. From October 2020 to February 2021, the PPHL performed 57,396 tests, observing a 25.6% positivity ratio. A significant proportion of these positive cases were attributed to the Alpha variant. To specifically detect this VOC, the PPHL ingeniously developed an in-house dropout PCR assay, specifically targeting the 69/70 deletion in the spike (S) gene. As shown in Fig. 2, two bands of 280 bp and 158 bp represented wildtype strains while those lanes with only the 158 bp band was due to the “dropout” or 69/70 deletion. A 100 bp DNA ladder was added for band size reference. From 100 positive samples tested during this wave, 55% (*n* = 55) had the 69/70 deletion and were therefore assigned as Alpha variant.

### Third wave and delta variant emergence (February–June 2021)

The third wave had co-circulation of Alpha and Delta (B.1.617.2) variants. The period between February and June 2021 marked Pakistan’s third COVID-19 wave. During this time, the PPHL performed 24,267 tests, with 5,016 positive SARS-CoV-2 cases (20.7% positivity). Genomic surveillance through RT-PCR assays indicated that most samples from this period were positive for either the Alpha (*n* = 271) or Delta variant (*n* = 482). The emergence of Delta, known for its increased transmissibility and often higher severity, significantly impacted case numbers and healthcare burden across Pakistan.

### Fourth wave (July–December 2021)

From July to December 2021, Pakistan experienced another surge in COVID-19 cases, associated with the Delta variant. The PPHL conducted 25,745 tests during this period, with 6,090 positive samples, a positivity rate of 23.7%. During this wave, the variants identified by RT-PCR were either Delta (*n* = 201) or Omicron (*n* = 172), with the first case of Omicron identified on December 25, 2021.

### Omicron wave (December 2021–early 2022)

The arrival of the Omicron variant in December 2021 ushered in yet another sharp increase in reported cases, initiating Pakistan’s fifth wave of pandemic. Omicron, like Alpha, also harboured the 69/70 deletion, making it detectable via the previously established dropout PCR assay. During this wave, the PPHL performed 9,265 tests, with 2,257 positive results. A combination of dropout PCR and commercially available RT-PCR kits for variant detection showed that Omicron was most dominant (*n* = 413), and a few cases of Delta (*n* = 31) were also reported.

**Fig. 3:**
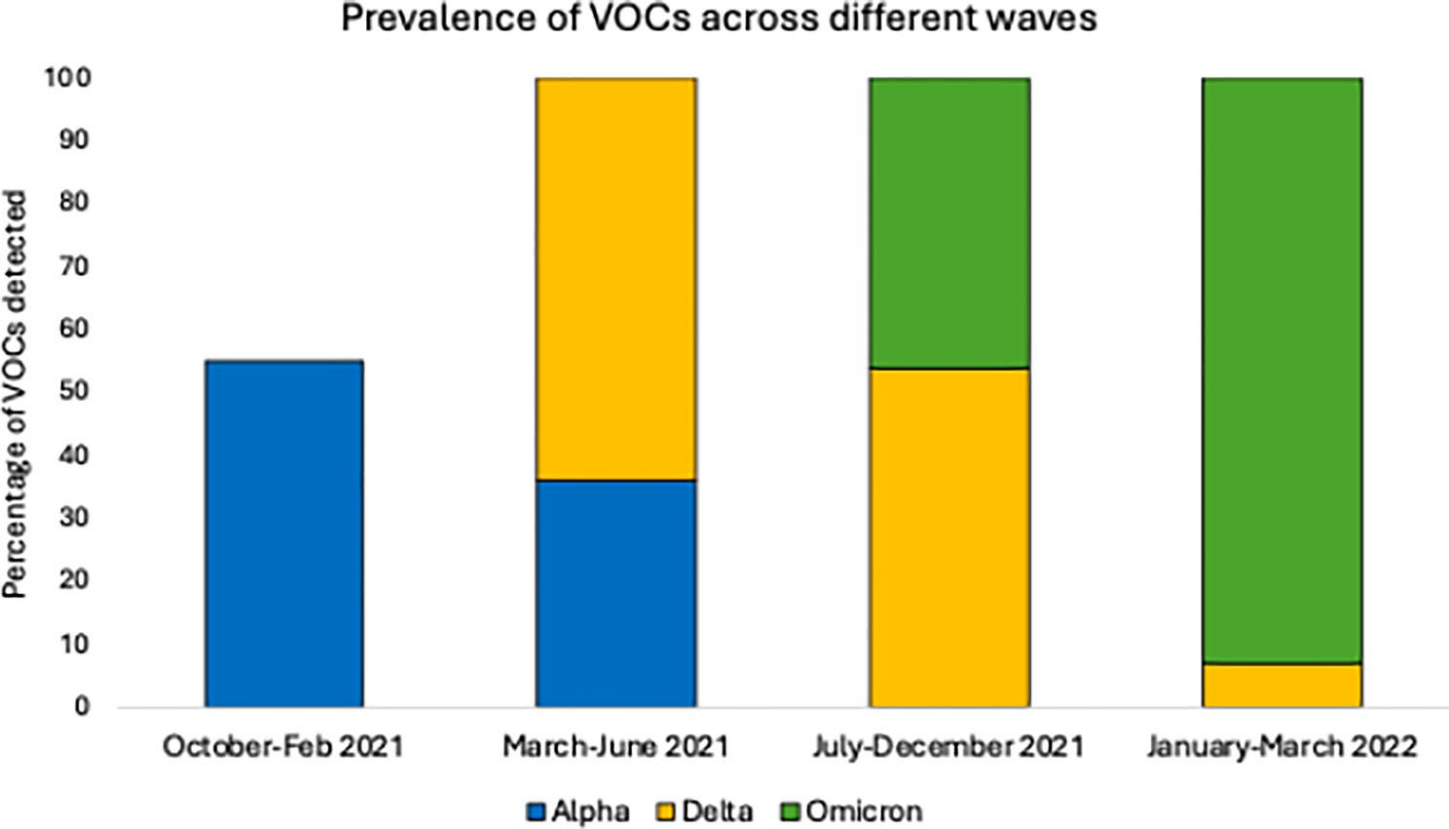
Percentage of VOCs detected throughout the second (October-February 2021), third (March-June 2021), fourth (July-December 2021) and fifth (January-March 2022) waves

The frequency of each VOC observed during the four major waves is shown in Fig. 3. From October to February 2021, 55% Alpha cases were reported, followed by 36% Alpha and 64% Delta cases between March and June 2021. Between the July-December 2021 wave, 54% Delta and 46% Omicron cases were reported while in the final January-March 2022 wave, 7% Delta and 93% Omicron cases were reported.

### May 2025 resurgence of COVID-19 cases

Despite the end of COVID-19 pandemic, continuous testing of suspected samples was conducted in PPHL. This continuous surveillance showed a sudden increase in COVID-19 cases in May 2025, when positive cases were observed globally in several other countries, including the USA, Singapore, Thailand, China, and India. Between May and June 2025, the PPHL conducted 666 tests, with 96 positive cases reported.

### Whole genome sequencing

In efforts to enhance genomic surveillance, a subset of SARS-CoV-2 positive samples during each of these waves from 2021 to 2025 were sequenced using platforms like ONT and IonTorrent. Between June 2021 and June 2025, 124 samples were successfully sequenced and submitted to the EpiCov GISAID database.

When sequencing first began, it was amidst the fourth wave, and between June and early December 2021 (before arrival of Omicron), 14 samples were sequenced, all of which were Delta variant with lineages B.1.617.2 (*n* = 6), AY.127 (*n* = 4), AY.126 (*n* = 2), and AY.108 (*n* = 2). During the fifth wave, further 22 samples were sequenced that revealed 19 Omicron lineages including BA.1 (*n* = 10), BA.2 (*n* = 1), and BA.5.2 (*n* = 8). 2 sequences belonged to Delta variant, lineage AY.127, while one sequence was not assigned a VOC and was declared ancestral-like (B). From late 2022 till early 2024, 67 more samples were sequenced and were analysed as XBB and its sublineages (*n* = 27), JN.1 (*n* = 21), BQ (*n* = 5), BA.5 (*n* = 4), FL recombinants (*n* = 4), and others including GW, GK, DN, XCC, and BA.2.86.1 (*n* = 6). The first case of JN.1, that remained the most dominant Omicron subvariant, was from a sample collected on January 4, 2024.

**Fig. 4:**
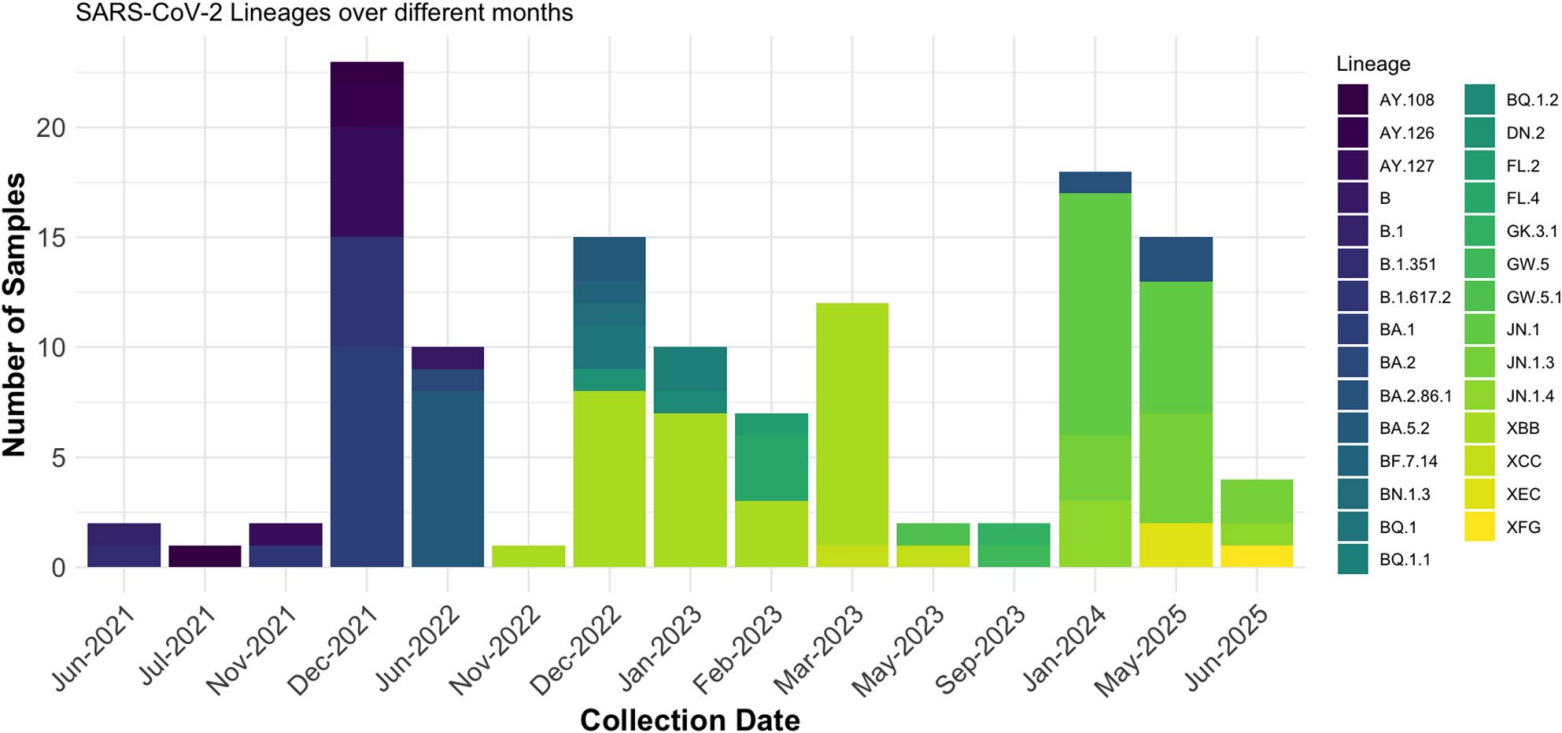
Frequency of different SARS-CoV-2 lineages that were identified from samples sequenced between June 2021 and June 2025

During the recent outbreak in May 2025, 19 samples were sequenced and all of them were either characterized as JN.1 and its sublineages (*n* = 14) or recombinants including XFG and XEC (*n* = 3). 2 sequences of BA.2.86 were also identified. Figure 4 shows frequency of each SARS-CoV-2 lineage, as identified by sequences uploaded on GISAID, along with sample collection date.

The dataset in Table 1 reflects the chronological emergence and dominance of key SARS-CoV-2 variants, transitioning from Delta sublineages (e.g., AY.108, AY.127) in 2021, to Omicron BA.1 and BA.2 in late 2021 and 2022, followed by the rise of BA.5, XBB sublineages, and BA.2.86 in 2023–2025. The Scorpio support values were high (≥ 0.90) across most lineages, indicating strong confidence in lineage assignments, while Scorpio conflict values remained low (≤ 0.05), suggesting minimal mutational discordance. A small number of sequences, particularly within the BA.2.86 and XBB.1.5 lineages, showed lower Scorpio call values (0.5–0.7), and were flagged as “Probable Omicron (Unassigned)”, reflecting emerging diversity or incomplete mutational profiles.

**Table 1:** SARS-CoV-2 lineages assigned by Pangolin with corresponding scorpio metrics

Collection date	Accession ID	Lineage	Scorpio call	Scorpio support	Scorpio conflict
01/06/2021	EPI_ISL_8317245	BA.5.2.1	Omicron (BA.5-like)	0.97	0.03
01/06/2021	EPI_ISL_8317246	BA.5.2	Omicron (BA.5-like)	0.97	0.03
04/07/2021	EPI_ISL_8317247	BA.5.2	Omicron (BA.5-like)	0.95	0.05
27/11/2021	EPI_ISL_8329848	BA.5.2	Omicron (BA.5-like)	0.97	0.03
28/11/2021	EPI_ISL_8329847	BA.5.2	Omicron (BA.5-like)	0.95	0.05
09/12/2021	EPI_ISL_7861807	BA.5.2	Omicron (BA.5-like)	0.95	0.03
10/12/2021	EPI_ISL_8170850	BA.2	Omicron (BA.2-like)	0.98	0.02
12/12/2021	EPI_ISL_8170852	BA.5.2	Omicron (BA.5-like)	0.98	0.02
13/12/2021	EPI_ISL_8170853	BA.5.2	Omicron (BA.5-like)	0.97	0.03
15/12/2021	EPI_ISL_8170855	XBB.1	Omicron (XBB-like)	0.84	0.01
16/12/2021	EPI_ISL_8170856	XBB.1	Omicron (XBB-like)	0.85	0.01
17/12/2021	EPI_ISL_8170857	XBB	Omicron (XBB.1-like)	0.86	0.05
22/12/2021	EPI_ISL_8329849	BA.5.2.49	Omicron (BA.5-like)	0.93	0.05
23/12/2021	EPI_ISL_8487235	BQ.1.1	Omicron (BA.5-like)	0.92	0.03
25/12/2021	EPI_ISL_8329837	XBB.1	Omicron (XBB-like)	0.88	0.01
25/12/2021	EPI_ISL_8329838	BQ.1.1	Omicron (BA.5-like)	0.95	0.03
25/12/2021	EPI_ISL_8329839	XBB.1.5.24	Omicron (XBB-like)	0.94	0.01
25/12/2021	EPI_ISL_8329840	XBB.1	Omicron (XBB-like)	0.77	0.01
25/12/2021	EPI_ISL_8329841	BN.1.3	Omicron (BA.2-like)	0.92	0.02
25/12/2021	EPI_ISL_8329842	XBB.1.9.2	Omicron (XBB-like)	0.88	0.01
25/12/2021	EPI_ISL_8329843	BA.5.2.48	Omicron (BA.5-like)	0.92	0.03
25/12/2021	EPI_ISL_8329844	XBB.1.9.1	Omicron (XBB-like)	0.88	0.01
25/12/2021	EPI_ISL_8329845	XBB.1	Omicron (XBB-like)	0.95	0.01
25/12/2021	EPI_ISL_8486876	XBB.1.37	Omicron (XBB-like)	0.95	0.01
27/12/2021	EPI_ISL_8329846	BQ.1	Omicron (BA.2-like)	0.84	0.08
27/12/2021	EPI_ISL_8329850	DN.2	Omicron (BA.5-like)	0.92	0.03
15/06/2022	EPI_ISL_14441437	BF.7.14	Omicron (BA.5-like)	0.92	0.05
16/06/2022	EPI_ISL_14441438	BQ.1.1	Omicron (BA.5-like)	0.95	0.03
23/06/2022	EPI_ISL_14441436	XBB.1.5	Omicron (XBB-like)	0.94	0.01
24/06/2022	EPI_ISL_14441435	BQ.1.2	Omicron (BA.5-like)	0.95	0.03
24/06/2022	EPI_ISL_14441439	XBB.1.5	Omicron (XBB-like)	0.86	0.01
25/06/2022	EPI_ISL_14441440	XBB.1.11	Omicron (XBB-like)	0.73	0
26/06/2022	EPI_ISL_14441434	FL.4	Omicron (XBB.1-like)	0.7	0.01
27/06/2022	EPI_ISL_14441431	XBB.1.19.1	Omicron (XBB-like)	0.73	0
27/06/2022	EPI_ISL_14441432	XBB.1.19.1	Omicron (XBB-like)	0.7	0
27/06/2022	EPI_ISL_14441433	XBB.1.9	Omicron (XBB-like)	0.73	0
29/11/2022	EPI_ISL_17445917	XBB.1.9.2	Omicron (XBB.1-like)	0.94	0.01
01/12/2022	EPI_ISL_17445535	FL.4	Omicron (XBB-like)	0.73	0
04/12/2022	EPI_ISL_17445924	XBB.1.5	Omicron (XBB-like)	0.93	0.04
11/12/2022	EPI_ISL_17445532	XBB.1.19	Omicron (XBB-like)	0.73	0
11/12/2022	EPI_ISL_17445923	XBB.1.19.1	Omicron (XBB-like)	0.7	0
15/12/2022	EPI_ISL_17445534	XBB.1	Omicron (XBB-like)	0.73	0
15/12/2022	EPI_ISL_17445920	XBB.1.19	Omicron (XBB-like)	0.73	0.01
15/12/2022	EPI_ISL_17445926	XBB.1.5	Probable Omicron (Unassigned)	0.62	0
21/12/2022	EPI_ISL_17445925	FL.2	Omicron (XBB-like)	0.72	0.01
22/12/2022	EPI_ISL_17445536	XBB.1.5.24	Omicron (XBB-like)	0.83	0.01
23/12/2022	EPI_ISL_17445918	XBB.1.9.2	Omicron (XBB-like)	0.94	0.01
24/12/2022	EPI_ISL_17445921	XBB.1	Omicron (XBB-like)	0.69	0
25/12/2022	EPI_ISL_17445922	XCC	Omicron (XBB-like)	0.73	0
26/12/2022	EPI_ISL_17445919	XBB.1.19.1	Omicron (XBB-like)	0.78	0
29/12/2022	EPI_ISL_17445531	XBB.1.19.1	Omicron (BA.3-like)	0.58	0.04
29/12/2022	EPI_ISL_17445533	XBB.1	Omicron (XBB-like)	0.72	0.01
01/01/2023	EPI_ISL_17445927	XBB.1.5.24	Omicron (XBB-like)	0.79	0.04
02/01/2023	EPI_ISL_17445928	FL.4	Omicron (XBB-like)	0.78	0.01
03/01/2023	EPI_ISL_17445929	BA.2.86	Omicron (BA.2-like)	0.92	0.03
03/01/2023	EPI_ISL_17445930	BA.2	Omicron (BA.2-like)	0.76	0.03
07/01/2023	EPI_ISL_17445538	GK.3.1	Omicron (XBB.1.5-like)	0.93	0.03
07/01/2023	EPI_ISL_17474688	GW.5	Omicron (XBB-like)	0.94	0.01
07/01/2023	EPI_ISL_17474690	GW.5	Omicron (XBB-like)	0.94	0.01
08/01/2023	EPI_ISL_17445537	XCC	Omicron (XBB.1-like)	0.93	0.04
16/01/2023	EPI_ISL_17474674	BA.2	Omicron (BA.2-like)	0.89	0.06
24/01/2023	EPI_ISL_17474689	BA.2.86	Probable Omicron (Unassigned)	0.62	0
02/02/2023	EPI_ISL_17474686	BA.2	Probable Omicron (Unassigned)	0.53	0.03
13/02/2023	EPI_ISL_17474695	BA.2.86	Omicron (BA.2-like)	0.81	0.02
13/02/2023	EPI_ISL_17474696	BA.2.86	Probable Omicron (Unassigned)	0.62	0
16/02/2023	EPI_ISL_17474680	BA.2.86	Omicron (BA.2-like)	0.89	0.05
20/02/2023	EPI_ISL_17474685	BA.2.86	Omicron (BA.2-like)	0.82	0.03
21/02/2023	EPI_ISL_17474687	BA.2.86	Probable Omicron (Unassigned)	0.62	0
25/02/2023	EPI_ISL_17474675	BA.2.86	Omicron (BA.2-like)	0.79	0.02
03/03/2023	EPI_ISL_17474679	BA.2.86	Probable Omicron (Unassigned)	0.62	0
13/03/2023	EPI_ISL_17474682	BA.2.86	Probable Omicron (Unassigned)	0.66	0
14/03/2023	EPI_ISL_17474677	BA.2	Probable Omicron (Unassigned)	0.66	0.03
14/03/2023	EPI_ISL_17474683	BA.2.86	Omicron (BA.2-like)	0.85	0.03
14/03/2023	EPI_ISL_17474691	BA.2.86	Omicron (BA.2-like)	0.84	0.03
16/03/2023	EPI_ISL_17474678	BA.2.86	Omicron (BA.2-like)	0.87	0.05
16/03/2023	EPI_ISL_17474692	BA.2	Omicron (BA.2-like)	0.85	0.03
20/03/2023	EPI_ISL_17474693	BA.2.86	Omicron (BA.2-like)	0.77	0.02
20/03/2023	EPI_ISL_17474694	BA.2.86	Omicron (BA.2-like)	0.9	0.02
21/03/2023	EPI_ISL_17474676	BA.2.86	Omicron (BA.2-like)	0.9	0.03
21/03/2023	EPI_ISL_17474681	BA.2.86	Probable Omicron (Unassigned)	0.66	0
24/03/2023	EPI_ISL_17474684	BA.2.86	Omicron (BA.2-like)	0.77	0.03
01/05/2023	EPI_ISL_18788093	BA.2.86	Omicron (Unassigned)	0.81	0
09/05/2023	EPI_ISL_18788092	BA.2.86	Omicron (BA.2-like)	0.84	0.03
06/09/2023	EPI_ISL_18788090	BA.2.86	Omicron (BA.2-like)	0.85	0.03
06/09/2023	EPI_ISL_18788091	BA.2.86	Probable Omicron (Unassigned)	0.5	0
04/01/2024	EPI_ISL_18788088	BA.2.86	Probable Omicron (Unassigned)	0.5	0
06/01/2024	EPI_ISL_18788089	BA.2.86	Omicron (BA.2-like)	0.92	0.03
07/01/2024	EPI_ISL_18788094	BA.2.86	Omicron (BA.2-like)	0.92	0.03
11/01/2024	EPI_ISL_18831496	BA.2.86	Omicron (BA.2-like)	0.81	0.02
14/01/2024	EPI_ISL_18831497	BA.2.86	Omicron (BA.2-like)	0.87	0.02
15/01/2024	EPI_ISL_18831498	BA.2.86	Omicron (BA.2-like)	0.81	0.03
15/01/2024	EPI_ISL_18831499	BA.2.86	Omicron (BA.2-like)	0.82	0.03
17/01/2024	EPI_ISL_18831500	BA.2.86	Omicron (BA.2-like)	0.92	0.03
17/01/2024	EPI_ISL_18831501	BA.2.86	Omicron (BA.2-like)	0.9	0.03
17/01/2024	EPI_ISL_18831502	BA.2.86	Probable Omicron (Unassigned)	0.66	0
17/01/2024	EPI_ISL_18831503	AY.108	Delta (B.1.617.2-like)	0.77	0.23
17/01/2024	EPI_ISL_18831504	AY.127	Delta (B.1.617.2-like)	0.92	0
17/01/2024	EPI_ISL_18831505	AY.127	Delta (B.1.617.2-like)	0.85	0.08
18/01/2024	EPI_ISL_18831506	AY.126	Delta (B.1.617.2-like)	1	0
18/01/2024	EPI_ISL_18831507	B.1.617.2	Delta (B.1.617.2-like)	1	0
18/01/2024	EPI_ISL_18831508	AY.108	Delta (B.1.617.2-like)	0.92	0
19/01/2024	EPI_ISL_18831509	AY.126	Delta (B.1.617.2-like)	0.92	0
21/01/2024	EPI_ISL_18831510	B.1.617.2	Delta (B.1.617.2-like)	0.92	0
05/05/2025	EPI_ISL_19882295	B.1.351	Beta (B.1.351-like)	0.86	0
08/05/2025	EPI_ISL_19882293	B.1			
08/05/2025	EPI_ISL_19882301	AY.108	Delta (B.1.617.2-like)	0.92	0.08
12/05/2025	EPI_ISL_19882299	BA.1.1	Omicron (BA.1-like)	0.74	0
13/05/2025	EPI_ISL_19882292	BA.1.1	Omicron (BA.1-like)	0.71	0
14/05/2025	EPI_ISL_19882294	BA.1.1	Omicron (BA.1-like)	0.74	0
15/05/2025	EPI_ISL_19882300	AY.127	Delta (B.1.617.2-like)	0.85	0.08
17/05/2025	EPI_ISL_19882291	BA.1	Omicron (BA.1-like)	0.74	0
17/05/2025	EPI_ISL_19882296	BA.1	Omicron (BA.1-like)	0.74	0.02
17/05/2025	EPI_ISL_19882298	BA.1.1	Omicron (BA.1-like)	0.74	0
19/05/2025	EPI_ISL_19882297	BA.1.1	Omicron (BA.1-like)	0.78	0.07
28/05/2025	EPI_ISL_20055948	BA.1.1	Omicron (BA.1-like)	0.76	0.1
30/05/2025	EPI_ISL_20055951	BA.1	Probable Omicron (Unassigned)	0.66	0.19
31/05/2025	EPI_ISL_20055952	AY.127	Delta (B.1.617.2-like)	0.92	0
31/05/2025	EPI_ISL_20055953	B.1.617.2	Delta (B.1.617.2-like)	0.85	0
02/06/2025	EPI_ISL_20055954	AY.127	Delta (B.1.617.2-like)	0.92	0
03/06/2025	EPI_ISL_20055949	AY.127	Delta (B.1.617.2-like)	0.92	0
06/06/2025	EPI_ISL_20055950	BA.1.1	Omicron (BA.1-like)	0.79	0.07
09/06/2025	EPI_ISL_20055955	B.1.617.2	Delta (B.1.617.2-like)	0.92	0

This table presents SARS-CoV-2 lineages assigned using the Pangolin tool for sequences collected in Karachi between June 2021 and June 2025. Each sequence was analyzed to determine its lineage and associated mutation profile using Scorpio, which reports a “Scorpio call” (defining variant designation), “Scorpio support” (number of defining mutations detected), and “Scorpio conflict” (number of conflicting mutations present)

### Phylogenetic analysis

**Fig. 5:**
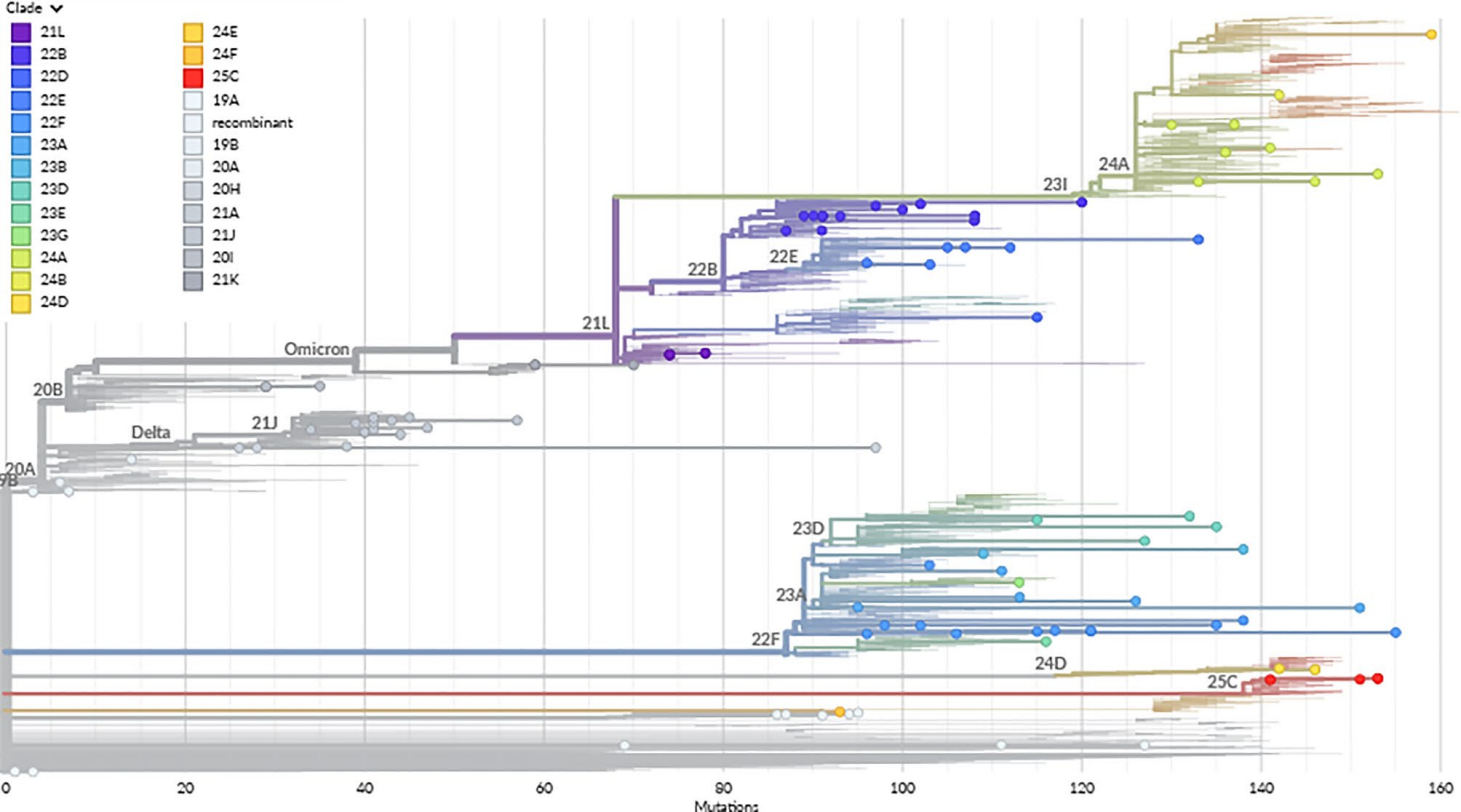
Phylogenetic tree constructed by Nextclade using 50 sequences obtained from the study and 46 global sequences uploaded on GISAID. Nextclade Pango lineages are assigned to each clade in the tree

**Fig. 6:**
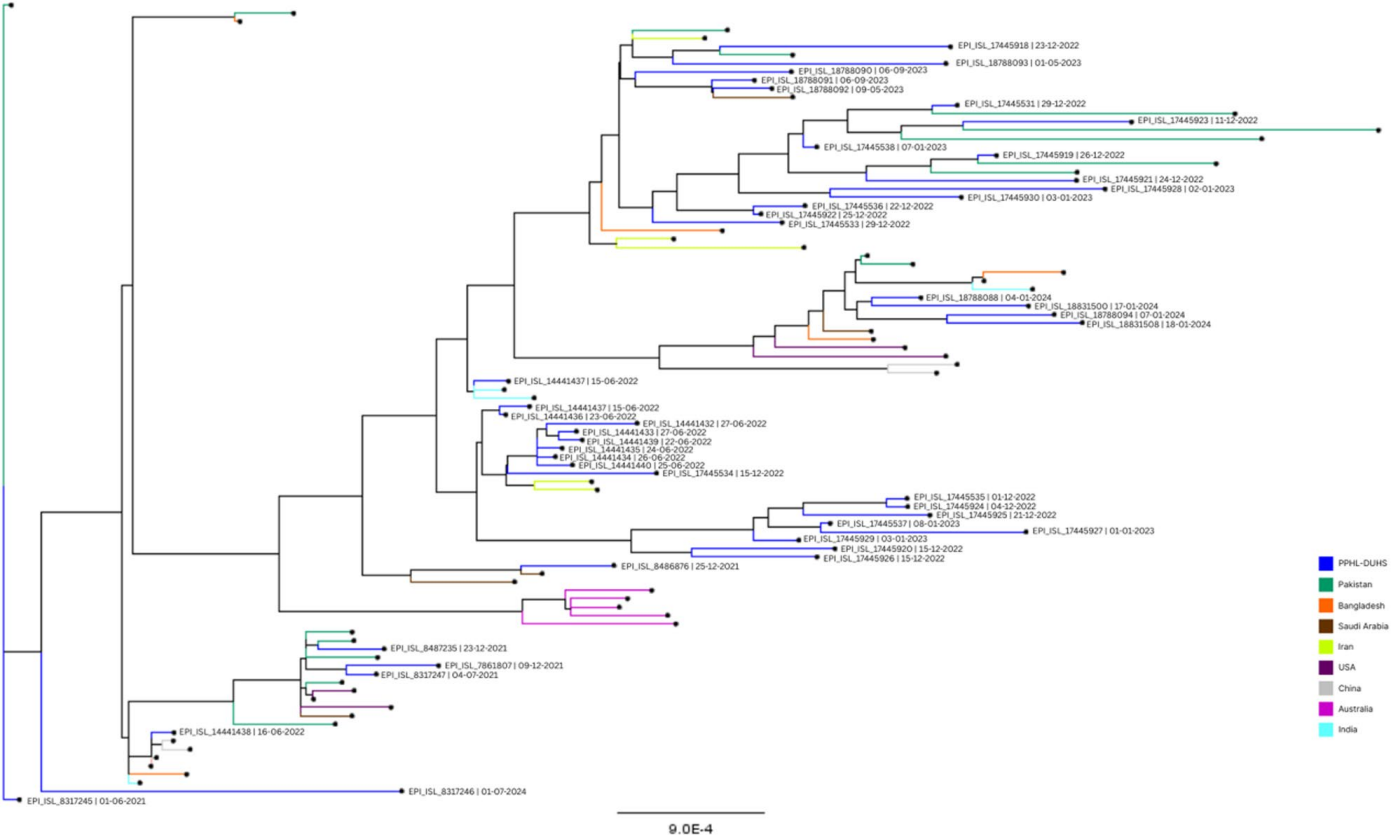
A maximum likelihood phylogenetic inference using IQ-TREE was constructed using Galaxy and viewed on FigTree. Sequences from Pakistan, Bangladesh, Saudi Arabia, Iran, USA, China, Australia and India were downloaded from GISAID for comparison

Phylogenetic trees were constructed using Nextclade and IQ-TREE, as shown in Figs. 5 and 6, respectively. The tree in Fig. 6 shows that PPHL-DUHS sequences (blue) do not cluster tightly in a single clade, which suggests multiple introductions or sustained evolution within Pakistan. Some PPHL-DUHS sequences from late 2022 and early 2023 appear to cluster together, indicating local circulation. Others are more distantly related, hinting at independent origins or introductions linked to travel or regional lineage mixing. There are visible groupings of PPHL-DUHS samples with sequences from neighbouring countries like Iran, India, and Bangladesh, suggesting regional transmission chains or shared ancestry. A few recent PPHL-DUHS samples also sit near international sequences from Australia, Saudi Arabia, and even the US (dark purple), implying more recent global connections. The presence of recent PPHL-DUHS samples from 2024 to 2025 toward terminal branches of the tree hints at novel evolutionary paths, local sub-lineages that deserve further analysis. This might be especially relevant for assessing mutations associated with immune escape or altered transmissibility. Genetic Diversity within PPHL-DUHS Samples indicates ongoing local transmission, with multiple introduction events and mutation accumulation, reflecting different waves (Delta, Omicron, etc.).

**Fig. 7:**
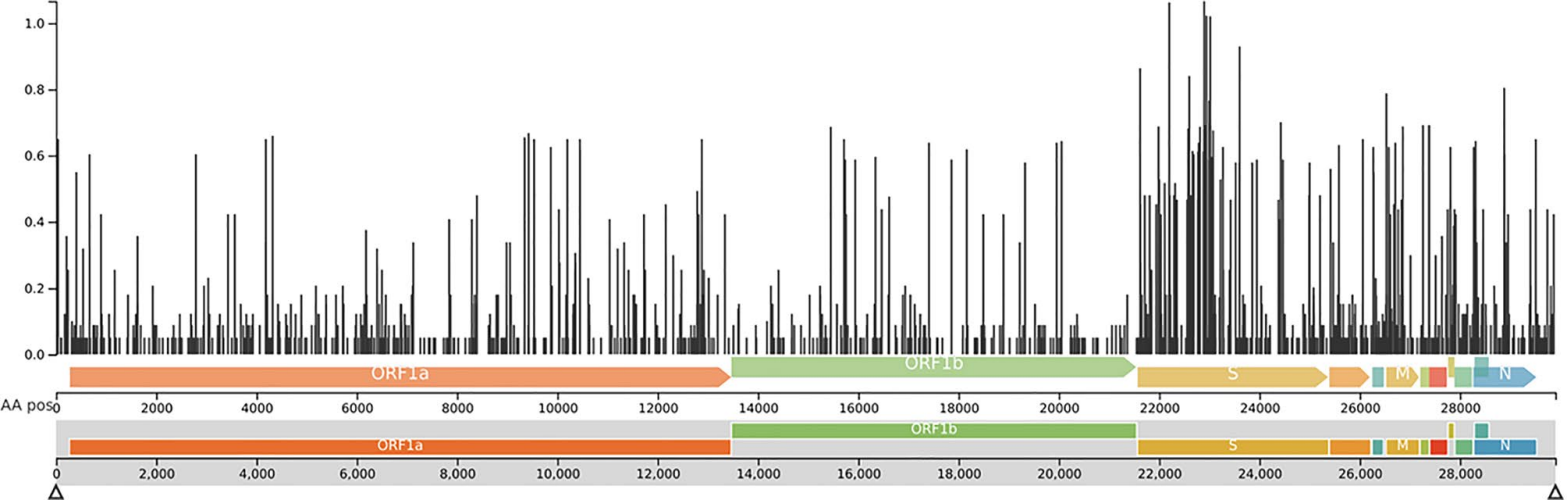
Genetic diversity across the SARS-CoV-2 genome as analysed by Nextclade. The x-axis represents different regions of the viral genome, while the y-axis indicates the normalized Shannon entropy

A Nextclade analysis was performed on the 116 SARS-CoV-2 genome sequences, representing multiple clades from Delta (21 J), Omicron (21 K, 22B, 22E, 22 F, 23D, 24 A, 24 F) to earlier lineages such as Beta (20 H) and ancestral clade 19 A. Most sequences exhibited high genome coverage (typically ≥ 95%), indicating good-quality assemblies. Most Omicron sequences, especially clades 21 K and 24 A, showed a high number of insertions (ranging from 10 to > 200) and frameshifts (up to 48 in some BA.2.86-related samples), consistent with the known rapid evolution and structural plasticity of this variant. In contrast, Delta (21 J) and Beta (20 H) sequences displayed fewer or no insertions and minimal frameshift mutations. Notably, some recent Omicron sequences from 2024 to 2025, particularly within clades 24 A and 24 F, showed exceptionally high mutational burdens. A few recombinant clades were also identified, further highlighting ongoing viral evolution. These results are shown in Table 2.

**Table 2:** Nextclade analysis of SARS-CoV-2 sequences showing WHO clades, coverage, insertions, and frameshifts

ACCESSION ID	CLADE	CLADE WHO	COVERAGE (%)	TOTAL INSERTIONS	TOTAL FRAMESHIFTS
EPI_ISL_14441431	22B	Omicron	99.5	0	0
EPI_ISL_14441432	22B	Omicron	99.7	0	0
EPI_ISL_14441433	22B	Omicron	99.6	0	0
EPI_ISL_14441434	22B	Omicron	99.6	0	0
EPI_ISL_14441435	22B	Omicron	99.6	0	0
EPI_ISL_14441436	22B	Omicron	99.6	0	0
EPI_ISL_14441437	21 L	Omicron	99.7	0	0
EPI_ISL_14441438	19 A		99.7	0	0
EPI_ISL_14441439	22B	Omicron	99.6	0	0
EPI_ISL_14441440	22B	Omicron	99.6	0	0
EPI_ISL_17445531	22 F		96.4	0	0
EPI_ISL_17445532	22 F		94.0	9	0
EPI_ISL_17445533	22 F		96.0	1	0
EPI_ISL_17445534	22B	Omicron	97.9	1	0
EPI_ISL_17445535	22E	Omicron	97.0	0	0
EPI_ISL_17445536	22 F		96.3	0	0
EPI_ISL_17445537	22E	Omicron	96.9	0	0
EPI_ISL_17445538	23 A		97.8	0	0
EPI_ISL_17445917	22 F		94.6	2	1
EPI_ISL_17445918	22D	Omicron	94.9	1	1
EPI_ISL_17445919	23D		95.5	1	2
EPI_ISL_17445920	22B	Omicron	98.8	14	2
EPI_ISL_17445921	23D		95.7	3	2
EPI_ISL_17445922	22 F		97.9	1	1
EPI_ISL_17445923	22 F		98.3	1	2
EPI_ISL_17445924	22E	Omicron	96.7	6	2
EPI_ISL_17445925	22E	Omicron	97.5	2	3
EPI_ISL_17445926	22B	Omicron	96.3	1	1
EPI_ISL_17445927	22E	Omicron	97.5	3	3
EPI_ISL_17445928	23 A		97.1	8	1
EPI_ISL_17445929	22E	Omicron	97.9	0	1
EPI_ISL_17445930	23 A		95.5	9	2
EPI_ISL_17474674	22 F		95.0	1	1
EPI_ISL_17474675	23D		94.7	4	3
EPI_ISL_17474676	22 F		93.6	1	2
EPI_ISL_17474677	22 F		92.5	0	2
EPI_ISL_17474678	23D		92.7	7	3
EPI_ISL_17474679	23D		97.0	9	2
EPI_ISL_17474680	23D		93.6	14	3
EPI_ISL_17474681	23 A		98.1	2	1
EPI_ISL_17474682	22 F		93.2	2	2
EPI_ISL_17474683	22 F		91.9	4	2
EPI_ISL_17474684	22 F		93.1	0	1
EPI_ISL_17474685	22 F		93.9	15	4
EPI_ISL_17474686	23 A		81.3	12	6
EPI_ISL_17474687	23D		92.8	15	4
EPI_ISL_17474688	23 A		94.6	2	2
EPI_ISL_17474689	23D		97.0	4	2
EPI_ISL_17474690	22 F		89.5	1	1
EPI_ISL_17474691	recombinant	recombinant	94.3	0	0
EPI_ISL_17474692	22 F		95.0	4	4
EPI_ISL_17474693	22 F		85.5	4	2
EPI_ISL_17474694	22 F		94.4	4	2
EPI_ISL_17474695	23 A		94.5	17	3
EPI_ISL_17474696	23D		93.8	3	1
EPI_ISL_18788088	24 A	Omicron	99.7	12	2
EPI_ISL_18788089	24 A	Omicron	98.8	39	8
EPI_ISL_18788090	23G		99.7	0	2
EPI_ISL_18788091	22 F		99.6	0	2
EPI_ISL_18788092	22 F		99.5	0	1
EPI_ISL_18788093	recombinant	recombinant	99.5	26	6
EPI_ISL_18788094	24 A	Omicron	99.5	25	3
EPI_ISL_18831496	24 A	Omicron	99.5	16	12
EPI_ISL_18831497	22B	Omicron	98.9	70	46
EPI_ISL_18831498	24 A	Omicron	99.6	13	5
EPI_ISL_18831499	24 A	Omicron	99.5	113	23
EPI_ISL_18831500	24 A	Omicron	99.6	15	4
EPI_ISL_18831501	24 A	Omicron	99.5	17	2
EPI_ISL_18831502	24 A	Omicron	99.5	12	9
EPI_ISL_18831503	24 A	Omicron	99.7	24	8
EPI_ISL_18831504	24 A	Omicron	99.3	13	8
EPI_ISL_18831505	24 A	Omicron	99.0	13	5
EPI_ISL_18831506	24 A	Omicron	99.1	108	42
EPI_ISL_18831507	24 A	Omicron	99.5	17	6
EPI_ISL_18831508	24 A	Omicron	99.5	12	3
EPI_ISL_18831509	24 A	Omicron	99.5	12	7
EPI_ISL_18831510	24 A	Omicron	99.5	12	3
EPI_ISL_19882291	24 F		99.1	12	4
EPI_ISL_19882292	24 F		99.7	18	5
EPI_ISL_19882293	24 A	Omicron	99.8	12	6
EPI_ISL_19882294	24 F		81.5	29	7
EPI_ISL_19882295	24 A	Omicron	80.9	16	5
EPI_ISL_19882296	24 F		90.2	111	8
EPI_ISL_19882297	24 A	Omicron	98.1	566	6
EPI_ISL_19882298	24 A	Omicron	91.9	16	6
EPI_ISL_19882299	20B		98.6	98	48
EPI_ISL_19882300	20B		99.8	145	41
EPI_ISL_19882301	24 A	Omicron	95.8	0	5
EPI_ISL_7861807	21 J	Delta	94.8	8	2
EPI_ISL_8170850	21 J	Delta	99.3	17	5
EPI_ISL_8170851	21 J	Delta	99.2	17	5
EPI_ISL_8170852	21 J	Delta	99.5	25	5
EPI_ISL_8170853	21 J	Delta	99.8	17	5
EPI_ISL_8170854	21 J	Delta	61.6	101	17
EPI_ISL_8170855	21 J	Delta	95.1	27	7
EPI_ISL_8170856	21 J	Delta	97.7	18	6
EPI_ISL_8170857	21 J	Delta	97.6	17	5
EPI_ISL_8317245	20 H	Beta	99.7	0	0
EPI_ISL_8317246	20 H	Beta	97.0	23	3
EPI_ISL_8317247	21 J	Delta	99.7	0	0
EPI_ISL_8329837	21 K	Omicron	97.9	222	35
EPI_ISL_8329838	21 K	Omicron	93.5	217	33
EPI_ISL_8329839	21 K	Omicron	98.1	212	34
EPI_ISL_8329840	21 J	Delta	98.8	211	35
EPI_ISL_8329841	21 K	Omicron	97.7	214	35
EPI_ISL_8329842	21 K	Omicron	98.0	217	34
EPI_ISL_8329843	21 K	Omicron	98.1	217	36
EPI_ISL_8329844	21 K	Omicron	98.2	212	34
EPI_ISL_8329845	21 K	Omicron	98.2	221	37
EPI_ISL_8329846	21 K	Omicron	97.0	212	33
EPI_ISL_8329847	21 J	Delta	96.8	209	34
EPI_ISL_8329848	21 J	Delta	96.7	209	34
EPI_ISL_8329849	21 J	Delta	96.9	209	33
EPI_ISL_8329850	21 J	Delta	98.1	218	34
EPI_ISL_8486876	21 K	Omicron	98.5	10	1
EPI_ISL_8487235	21 J	Delta	99.0	0	0

This table summarizes the results of Nextclade analysis for SARS-CoV-2 sequences where each sequence was assigned a WHO clade based on phylogenetic placement. The table includes genome coverage (%), number of insertions, and frameshift mutations detected. High-quality sequences were defined as those with ≥ 95% coverage and no frameshifts

As shown in Fig. 7, a plot illustrating genetic diversity across the SARS-CoV-2 genome was generated using Nextclade. The highest degree of variability was observed in the *S* gene, with Shannon entropy reaching as high as 1.171.

## Discussion

### Overview of surveillance findings

Pakistan mirrored a global trajectory, experiencing five comparable waves of varying intensity and duration. Notably, each wave in Pakistan coincided with the emergence and widespread transmission of a specific VOC, or in some cases, with concurrent circulation of multiple variants [12]. These variants Alpha (B.1.1.7), Beta (B.1.351), Gamma (P.1), Delta (B.1.617.2), and Omicron (B.1.1.529 and its sublineages) were associated with increased transmissibility, partial immune escape, or altered disease severity, contributing to the successive surges in case numbers [13]. A total of 250 SARS-CoV-2 positive samples were tested using the in-house dropout PCR assay targeting the Δ69/70 deletion in the spike gene. Of the 100 samples analysed during the period of Alpha variant predominance, 55 (55%) showed dropout amplification, indicating the presence of the Δ69/70 deletion. During the subsequent Omicron emergence phase, 150 samples were tested, of which 113 (75.3%) exhibited the dropout pattern consistent with the deletion. To confirm assay specificity, a subset of the PCR-negative samples from both phases was subjected to Sanger sequencing. These were confirmed to be either wild-type strains or other variants not harbouring the Δ69/70 deletion.

### SARS-CoV-2 waves and variant circulation

The first wave (April–August 2020) was marked by the wild-type SARS-CoV-2 strain, with no formal variant tracking [14]. PPHL responded quickly by expanding molecular testing, achieving a 29.9% positivity rate. During the second wave (October 2020–February 2021), the Alpha variant emerged. In response, PPHL developed a cost-effective in-house dropout PCR assay targeting the 69/70 deletion in the spike gene. This assay, validated through Sanger sequencing, proved effective in detecting Alpha and later Omicron variants. The third wave (February–June 2021) saw co-circulation of Alpha and Delta variants [15]. Delta’s increased transmissibility significantly influenced case numbers and by the fourth wave (July–December 2021), Delta became dominant [16]. PPHL implemented WGS using the IonTorrent platform, identifying key Delta sublineages such as AY.127 and B.1.617.2, along with some Beta cases. The fifth wave (December 2021–early 2022) was driven by Omicron. Using both dropout PCR and the EscapePLEX RT-PCR kit, PPHL detected a dominant Omicron presence (78.3% of 575 samples tested), alongside Delta and Beta. Further sequencing revealed a wide range of Omicron subvariants and recombinant forms, including XBB.1.9.1 and BA.5.2 [17]. In May 2025, Pakistan experienced a resurgence linked to emerging global variants like JN.1 and BA.2.86.1. Of 545 samples tested, 96 were positive, and 11 were sequenced confirming the presence of these new lineages. This pattern demonstrates the ongoing evolution of SARS-CoV-2 and reinforces the need for vigilant genomic surveillance.

In the context of the evolving SARS-CoV-2 pandemic, Fig. 4 provides a visual representation of variant dynamics within a specific study duration spanning from December 2021 to June 2025 [18]. Distinct temporal patterns of variant prevalence are evident, with the early dominance of the Delta lineage (B.1.617.2) and the initial Omicron wave (BA.1) in December 2021 transitioning to the emergence and circulation of various Omicron sub-lineages, including BA.2 and BA.5.2 throughout 2022 and 2023. Notably, the Figure also highlights a significant shift in variant landscape by May 2025, where JN.1 exhibits the highest recorded frequency, alongside the presence of XEC, underscoring the continuous evolution and succession of SARS-CoV-2 strains. These findings align with global epidemiological trends where each successive wave was driven by variants with increased transmissibility and/or immune escape properties. Pakistan, like India, has experienced waves driven by major global VOCs and VOIs. The highly transmissible Delta variant, which was first detected in India and caused a devastating wave there, subsequently led to a significant surge in Pakistan [19]. Similarly, both countries have seen dominance shift to the numerous Omicron subvariants (e.g., BA.1, BA.2, XBB.1, and JN.1) in later phases of the pandemic. This pattern aligns with the finding from a global comparative analysis that Asia generally exhibited a downstream confluence of two large lineages diverging by two distinct clusters in its lineage evolution pattern, reflecting the complex, multi-lineage circulation seen in the region [20]. In contrast, another emerging variant, NB.1, while reported in India, has not been reported in Pakistan yet, highlighting regional differences in variant circulation and the ongoing need for vigilant genomic surveillance, a practice that advocates for to track and respond to emerging variants globally.

The demographic analysis showed a predominance of male patients (62.6%) and a mean age of 40.6 years, consistent with international patterns where older males with comorbidities were at higher risk for severe disease.

### Local diagnostic capacity and assay development

To enhance early variant detection, PPHL developed an in-house dropout PCR assay targeting the 69/70 deletion in the spike (S) gene. This deletion was a key mutational marker for Alpha and Omicron variants. The dropout assay correctly identified 55% of Alpha-positive and 75.3% of Omicron-positive samples, and its findings were validated using Sanger sequencing. This cost-effective method proved instrumental in rapidly identifying VOCs in a resource-limited setting. From July 2021 to May 2022, variant detection was further refined using the SNPsig EscapePLEX SARS-CoV-2 PCR kit. Of 575 tested samples, 1% were identified as Beta, 16.9% as Delta, and 78.3% as Omicron. These results underscore the swift dominance of Omicron and support global observations of its enhanced transmissibility.

### Genomic and phylogenetic analysis of viral evolution

To monitor viral evolution and identify emerging lineages, PPHL sequenced 124 SARS-CoV-2 genomes from 2021 to 2025 using platforms like IonTorrent and ONT. These sequences, uploaded to GISAID, were classified into multiple lineages as seen in the results, (Table 1), illustrating the transition from Delta sublineages (AY.127, AY.108) to Omicron subvariants (BA.1, BA.5.2, XBB.1.9.1) and eventually to emerging lineages like JN.1 and BA.2.86.1. The Scorpio support values confirmed high confidence in lineage assignment. Nextclade analysis further revealed detailed clade distributions and mutational complexity, as seen in the results (Table 2). Omicron-related clades (21 K, 22B, 24 A) exhibited the highest number of insertions and frameshifts, especially in the spike gene region, confirming Omicron’s extraordinary mutational plasticity.

After removing low-quality coverage sequences, 50 SARS-CoV-2 sequences from PPHL Sindh were used to generate a phylogenetic tree from Nextclade, as shown in Fig. 5. This shows the evolutionary relationships among different lineages of the SARS-CoV-2 virus. The horizontal axis represents the accumulated number of mutations, indicating genetic divergence from an ancestral strain, while the vertical axis implicitly categorises different Pango lineages and major clades (like 20 A, 21 J, 21 L, 22B, 22E, 23D, 23I, 24 A, GISAID clades representing temporal progression). Each coloured line and dot correspond to a specific SARS-CoV-2 lineage, as identified by the extensive legend on the left side of the image, which lists numerous variants including Delta (B.1.617.2), various Omicron sub-lineages (e.g., BA.1, BA.5.2, JN.1), and XBB recombinants. The results depicted in this tree illustrate the dynamic and continuous evolution of SARS-CoV-2 throughout the pandemic. We can observe distinct phases of viral dominance and diversification: a significant expansion is seen around the “21J” clade, dominated by the blue lines representing the Delta variant (B.1.617.2). This indicates a period where Delta became a prevalent strain, accumulating a moderate number of mutations. A major evolutionary jump is evident with the emergence of the “Omicron” clade, starting around “21L” and “22B.” This branch is characterised by an initial prominent lineage, BA.1 (teal), and then a rapid and extensive diversification into a multitude of sub-lineages. The density of branches and numerous colours beyond “22B” clearly show the vast array of Omicron sub-lineages that quickly evolved, including BA.5.2, BQ.1.1, BN.1.3, and particularly the large cluster of XBB variants (various reds, oranges, and purples) which show extensive further mutation and branching. This illustrates Omicron’s high transmissibility and immune-evasive capabilities, driving its rapid global spread and subsequent evolution into many descendants. Towards the rightmost part of the tree, representing the most recent evolution (around “23I” and “24A”), lineages like JN.1 (light green) show significant presence and continued mutation accumulation. This reflects the global shifts in dominant strains, with JN.1 being a prominent variant in late 2023 and early 2024. A distinct, long branch labelled “recombinant” at the bottom highlights instances where genetic material from different co-circulating lineages combined to form new variants.

Nextclade was also used to plot the genomic diversity of SARS-CoV-2 sequences as shown in Fig. 7. The most prominent peaks in diversity are concentrated in specific areas. Notably, there are very high peaks in the region corresponding to the Spike (S) gene (around 22,000 to 26,000 bp). This is a crucial finding because the Spike protein is the main target of the human immune system and is responsible for viral entry [21]. High diversity here reflects the virus’s ability to mutate its Spike protein to evade host immune responses (immune escape) and potentially alter its transmissibility. The peaks in the S gene are indicative of the numerous mutations observed in VOCs like Alpha, Delta, and especially Omicron, which have acquired significant changes in their spike proteins [22]. Noticeable peaks of diversity were also observed within the ORF1a and ORF1b regions [23]. These mutations can impact viral replication efficiency, proofreading, and interactions with host factors, and therefore require a more significant investigation and surveillance. There are also spikes of diversity in some of the smaller accessory ORFs and potentially even in the N (Nucleocapsid) gene towards the end of the genome [24]. These variations might contribute to altered viral fitness, pathogenicity, or immune evasion mechanisms not directly related to the spike protein. Conversely, many parts of the genome show very low bars, indicating highly conserved regions. These are typically genes or parts of genes that are essential for the virus’s basic functions (e.g., core replication machinery), where mutations would be detrimental to the virus’s survival or replication capacity.

The perception that COVID-19 has been fully eradicated is both misleading and potentially hazardous to public health efforts, particularly in densely populated, resource-constrained metropolises like Karachi. The detection of lineages in this study, particularly JN.1, which has been a dominant global strain in late 2023 and early 2024, underscores the ongoing need for vigilant genomic surveillance in Pakistan. While the immediate clinical impact of these newer variants might be milder for a vaccinated and previously infected population, their high transmissibility poses a continuous public health challenge. In neighbouring regions of Southeast Asia, including China, the NB.1.8.1 variant has recently surged, and sporadic cases have been detected across the United States. Although this variant has not yet been reported in Pakistan, the absence of evidence is not the evidence of absence. Enhanced surveillance, both in frequency and geographic coverage, remains essential to detect potential new introductions early and assess their epidemiological implications.

While global headlines may have shifted, the SARS-CoV-2 pathogen remains an active, evolving, and alarming presence. SARS-CoV-2, an RNA virus with a high mutation rate, has evolved rapidly due to widespread global transmission [25]. Successive VOCs have demonstrated new epidemiological and immunological profiles. By mid-2025, emerging lineages like NB.1.8.1 underscore the virus’s ongoing evolution [26]. Moreover, of particular concern is viral recombination, where co-infecting lineages exchange genetic material, potentially generating variants with increased transmissibility and immune evasion. Given Karachi’s dense population and global connectivity, the risk of such recombination is real and troubling.

### Challenges in COVID-19 diagnosis

The assumption that newer variants are becoming milder oversimplifies the threat. Spike protein mutations can weaken vaccine-induced immunity, while changes in viral tropism may allow broader tissue infection. Even a moderately pathogenic, highly transmissible variant could overwhelm healthcare systems, especially in vulnerable, under-resourced settings like Karachi. Mutations in conserved genomic regions could also affect diagnostic accuracy, masking the true burden of disease. Studies have highlighted that diagnostic tools like RT-PCR and more commonly used rapid antigen tests for screening, are also prone to false negatives, especially in early stage of infection [27]. Furthermore, diagnostic sensitivity can be compromised by emerging mutations in primer or probe-binding regions, as evidenced by the S-gene Target Failure (SGTF) in most diagnostic assays for COVID-19 during the Alpha and Omicron waves [28]. The clinical overlap with other respiratory infections also complicates differential diagnosis, particularly in under-resourced settings where confirmatory testing is often unavailable. This difficulty in diagnosis is further compounded by the evolving etiology of febrile illnesses, where infectious diseases remain the leading cause of fever of unknown origin (FUO) (59.6% of cases in China between 2013 and 2022), with respiratory infections being the most common specific cause (13.5%), underscoring the challenge in distinguishing COVID-19 from other respiratory pathogens [29]. When a COVID-19 diagnosis is made, the focus shifts to containment and recovery, with key public health recommendations including strict home quarantine, isolation in a separate and well-ventilated room, and wearing a mask when around others in the household. Symptomatic treatment, such as antipyretics for fever, is also advised for recovery at home [30].

### Vaccine development and effectiveness

A critical issue is vaccine inaccessibility and dependence on outdated formulations based on ancestral strains. As antigenic drift continues, existing vaccines offer diminishing protection, especially for high-risk groups [31]. The rollout of mass vaccination programs during the COVID-19 pandemic significantly reduced severe disease, hospitalizations and deaths, even amid the emergence of new variants. Recent findings show that despite high transmission rates of Omicron and its sublineages, severity among positive cases remained low due to high vaccination rates in the population [32]. Therefore, updated, multivalent vaccines must be made available to maintain efficacy and keep up with viral evolution. The development of Circular RNA (circRNA) vaccines represents a promising new avenue to address the limitations of conventional and even current linear mRNA vaccines, offering a platform for producing updated, multivalent vaccines with improved efficacy [33].

### Long COVID complications

Beyond acute infection surveillance, advanced molecular and sequencing tools have proven invaluable in investigating Long COVID, a condition characterized by persistent symptoms beyond the acute phase [34]. Technologies such as metagenomic sequencing and transcriptomics have enabled the detection of lingering viral RNA, immune dysregulation, and host response signatures in affected individuals. These sophisticated tools are vital for exploring the multi-system nature of Long COVID, which is increasingly recognized as a disease with a spectrum of pathology, involving damage to multiple organs like the heart, lungs, kidneys, and brain, even after mild infection and sometimes without characteristic symptoms of Long COVID. Furthermore, the molecular signatures detected are consistent with key pathogenic hypotheses, including the presence of persistent SARS-CoV-2 viral components, chronic inflammation, and an emergent autoimmune response, evidenced by the presence of various autoantibodies (e.g., antinuclear antibodies) that may contribute to the tissue injury and lasting symptoms [35, 36]. These findings underscore the potential of genomic tools not only for tracking variants but also for elucidating the pathophysiology and guiding treatment of Long COVID, an area that remains underexplored in Pakistan [37].

Effective pandemic response hinges on robust genomic surveillance, which remains limited in low-resource settings due to infrastructure, funding, and workforce gaps. This creates dangerous blind spots, delaying detection of new variants until they spread widely. To mitigate future waves, Pakistan must invest in local sequencing capacity, secure updated vaccines, and improve public health messaging. Strengthening diagnostic infrastructure and critical care systems is vital. Strategic, science-driven action, not complacency, is essential to navigate COVID-19’s unpredictable future and protect vulnerable populations like those in Karachi.

## Limitations

Although the genomic surveillance of SARS-CoV-2 provided crucial evidence, it is important to acknowledge the study’s limitations. First, only a small fraction of positive cases (124 out of 43,430) underwent whole genome sequencing, which may not fully capture the genetic diversity of circulating SARS-CoV-2 strains. Second, sequencing was preferentially performed on samples with low Ct values, introducing a potential bias toward higher viral load cases and limiting representativeness across the broader population. Finally, the study’s geographic and temporal scope was largely restricted to Karachi during the study period, which may not reflect the variant distribution across other regions of Pakistan. Despite these limitations, the findings provide important insights into the molecular epidemiology of SARS-CoV-2 in a major metropolitan center and establish a foundation for broader genomic surveillance in the country.

Additionally, the study’s geographic scope was largely confined to Karachi which may limit generalizability into variant dynamics across other regions.

## Conclusion

This five-year retrospective study conducted by PPHL, Sindh, highlights the necessity for sustained diagnostic capacity, molecular surveillance, and genomic monitoring to address the COVID-19 situation in Pakistan. The pandemic evolution in Pakistan followed the global patterns, with five major waves driven by evolving variants of concern, Alpha, Delta, and multiple Omicron sub-lineages. PPHL’s early adoption of in-house diagnostic reagents like real-time PCR variant assays and whole-genome sequencing enabled detection and monitoring of emerging lineages even in a resource-limited setting. The identification of new and recombined sublineages, including JN.1, BA.2.86.1, and XBB variants, underscores the ongoing diversification of SARS-CoV-2. Limited access to new vaccines, underdeveloped genomic surveillance capacity, and diagnostic problems further add to risks, particularly in highly populated urban settings like Karachi. The findings of this study emphasise the need for continued investment in genomic surveillance, next-generation vaccine universal access, and molecular diagnostics and bioinformatics capacity development. Public health systems must remain and continue to be alert, responsive, and science-driven if they are going to be in a position to mitigate the impact of subsequent surges. The COVID-19 pandemic is not finished; but instead remains a dynamic and evolving public health challenge. Active, concerted, and well-funded efforts must be employed to protect the vulnerable groups and prevent the reversal of progress made over the past five years in the face of continuing viral evolution.

## Supplementary Information

Below is the link to the electronic supplementary material.

Supplementary Material 1

## Data Availability

Accession IDs of all sequences generated in this study are provided within the Supplementary Table 1.

## Abbreviations

ACE2: Angiotensin-Converting Enzyme 2
CFR: Case Fatality Rate
DUHS: Dow University of Health Sciences
GISAID: Global Initiative on Sharing All Influenza Data
MAFFT: Multiple Alignment using Fast Fourier Transform
N: Nucleocapsid (protein)
NGS: Next-Generation Sequencing
ORF: Open Reading Frame
PPHL: Provincial Public Health Laboratory
RT-PCR: Reverse Transcription Polymerase Chain Reaction
S: Spike (protein)
SARS-CoV-2: Severe Acute Respiratory Syndrome Coronavirus 2
VOC: Variant of Concern
VTM: Viral Transport Medium
WGS: Whole Genome Sequencing
WHO: World Health Organization
