# Supplementary material for: Tracking the pandemic through molecular and sequencing tools: a story of SARS- CoV-2 over five years, lessons learned, and further directions
Source: BMC Infect Dis. 2025 Nov 25;25:1816. doi: 10.1186/s12879-025-12200-x (PMC12751935; doi:10.1186/s12879-025-12200-x)
Supplement: Supplementary file 1 — Supplementary Material 1 [file 12879_2025_12200_MOESM1_ESM.docx]

**Title: Tracking the Pandemic Through Molecular and Sequencing Tools: A Story of SARS-CoV-2 Over Five Years, Lessons Learned, and Further Directions**

**Authors:** Saeed Khan, Hafsa Faruqui, Maria Zahid, Sharjeel Chaudhry, Zaira Rehman, Hamza Noor, Manaal Naushad

**SUPPLEMENTARY FILE**

**[Uncropped image]**

**
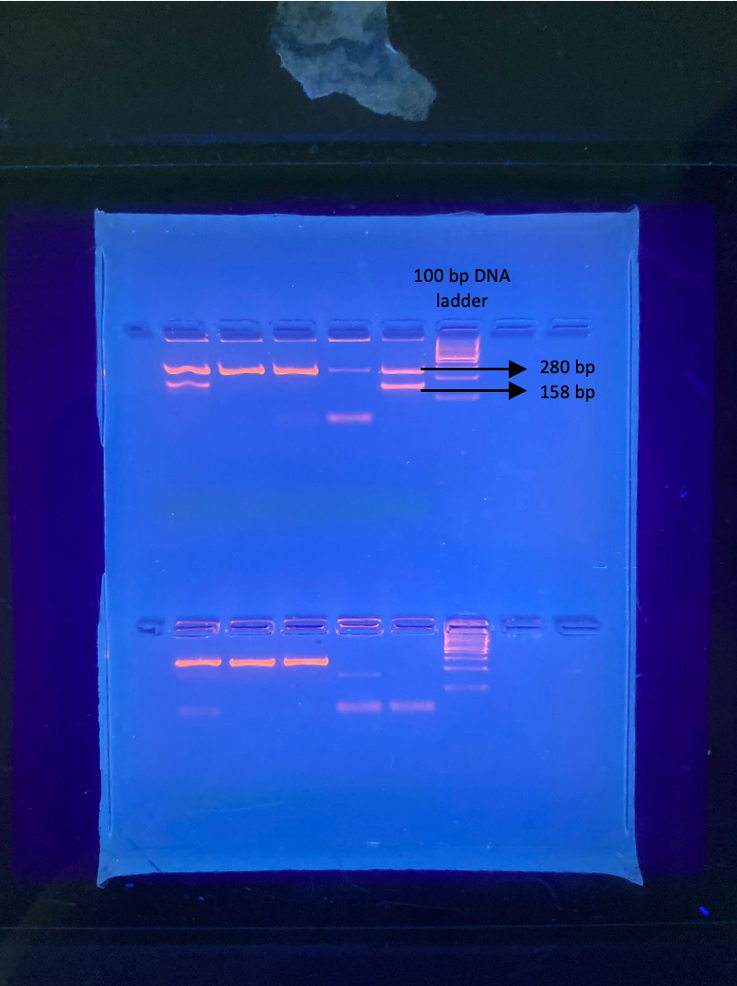
**

**Figure 1A:** 2% agarose gel electrophoresis showing bands of 280 bp and 158 bp for wildtype strains and a dropout of only 280 bp representing the 69/70 deletion. DNA marker ladder of 100 bp was run for reference size.

**Supplementary Table 1:** GISAID accession IDs of SARS-CoV-2 sequences submitted by PPHL-DUHS

| **PPHL-DUHS Accession IDs on GISAID** |
| --- |
| EPI_ISL_14441431 |
| EPI_ISL_14441432 |
| EPI_ISL_14441433 |
| EPI_ISL_14441434 |
| EPI_ISL_14441435 |
| EPI_ISL_14441436 |
| EPI_ISL_14441437 |
| EPI_ISL_14441438 |
| EPI_ISL_14441439 |
| EPI_ISL_14441440 |
| EPI_ISL_17445531 |
| EPI_ISL_17445532 |
| EPI_ISL_17445533 |
| EPI_ISL_17445534 |
| EPI_ISL_17445535 |
| EPI_ISL_17445536 |
| EPI_ISL_17445537 |
| EPI_ISL_17445538 |
| EPI_ISL_17445917 |
| EPI_ISL_17445918 |
| EPI_ISL_17445919 |
| EPI_ISL_17445920 |
| EPI_ISL_17445921 |
| EPI_ISL_17445922 |
| EPI_ISL_17445923 |
| EPI_ISL_17445924 |
| EPI_ISL_17445925 |
| EPI_ISL_17445926 |
| EPI_ISL_17445927 |
| EPI_ISL_17445928 |
| EPI_ISL_17445929 |
| EPI_ISL_17445930 |
| EPI_ISL_17474674 |
| EPI_ISL_17474675 |
| EPI_ISL_17474676 |
| EPI_ISL_17474677 |
| EPI_ISL_17474678 |
| EPI_ISL_17474679 |
| EPI_ISL_17474680 |
| EPI_ISL_17474681 |
| EPI_ISL_17474682 |
| EPI_ISL_17474683 |
| EPI_ISL_17474684 |
| EPI_ISL_17474685 |
| EPI_ISL_17474686 |
| EPI_ISL_17474687 |
| EPI_ISL_17474688 |
| EPI_ISL_17474689 |
| EPI_ISL_17474690 |
| EPI_ISL_17474691 |
| EPI_ISL_17474692 |
| EPI_ISL_17474693 |
| EPI_ISL_17474694 |
| EPI_ISL_17474695 |
| EPI_ISL_17474696 |
| EPI_ISL_18788088 |
| EPI_ISL_18788089 |
| EPI_ISL_18788090 |
| EPI_ISL_18788091 |
| EPI_ISL_18788092 |
| EPI_ISL_18788093 |
| EPI_ISL_18788094 |
| EPI_ISL_18831496 |
| EPI_ISL_18831497 |
| EPI_ISL_18831498 |
| EPI_ISL_18831499 |
| EPI_ISL_18831500 |
| EPI_ISL_18831501 |
| EPI_ISL_18831502 |
| EPI_ISL_18831503 |
| EPI_ISL_18831504 |
| EPI_ISL_18831505 |
| EPI_ISL_18831506 |
| EPI_ISL_18831507 |
| EPI_ISL_18831508 |
| EPI_ISL_18831509 |
| EPI_ISL_18831510 |
| EPI_ISL_19882291 |
| EPI_ISL_19882292 |
| EPI_ISL_19882293 |
| EPI_ISL_19882294 |
| EPI_ISL_19882295 |
| EPI_ISL_19882296 |
| EPI_ISL_19882297 |
| EPI_ISL_19882298 |
| EPI_ISL_19882299 |
| EPI_ISL_19882300 |
| EPI_ISL_19882301 |
| EPI_ISL_20055948 |
| EPI_ISL_20055949 |
| EPI_ISL_20055950 |
| EPI_ISL_20055951 |
| EPI_ISL_20055952 |
| EPI_ISL_20055953 |
| EPI_ISL_20055954 |
| EPI_ISL_20055955 |
| EPI_ISL_7861807 |
| EPI_ISL_8170850 |
| EPI_ISL_8170851 |
| EPI_ISL_8170852 |
| EPI_ISL_8170853 |
| EPI_ISL_8170854 |
| EPI_ISL_8170855 |
| EPI_ISL_8170856 |
| EPI_ISL_8170857 |
| EPI_ISL_8317245 |
| EPI_ISL_8317246 |
| EPI_ISL_8317247 |
| EPI_ISL_8329837 |
| EPI_ISL_8329838 |
| EPI_ISL_8329839 |
| EPI_ISL_8329840 |
| EPI_ISL_8329841 |
| EPI_ISL_8329842 |
| EPI_ISL_8329843 |
| EPI_ISL_8329844 |
| EPI_ISL_8329845 |
| EPI_ISL_8329846 |
| EPI_ISL_8329847 |
| EPI_ISL_8329848 |
| EPI_ISL_8329849 |
| EPI_ISL_8329850 |
| EPI_ISL_8486876 |
| EPI_ISL_8487235 |

This table lists the GISAID accession IDs corresponding to SARS-CoV-2 whole-genome sequences generated by PPHL, DUHS. These sequences were collected and processed between June 2021 and June 2025 as part of ongoing genomic surveillance efforts in Sindh. Metadata associated with each sequence (e.g., collection date, location, and host) can be accessed via the GISAID EpiCoV database.

**Supplementary Table 2:** GISAID accession IDs of SARS-CoV-2 sequences used in phylogenetic analysis

| Virus name | Accession ID |
| --- | --- |
| hCoV-19/Pakistan/DUHS09/2022 | EPI_ISL_14441431 |
| hCoV-19/Pakistan/DUHS10/2022 | EPI_ISL_14441432 |
| hCoV-19/Pakistan/DUHS11/2022 | EPI_ISL_14441433 |
| hCoV-19/Pakistan/DUHS13/2022 | EPI_ISL_14441434 |
| hCoV-19/Pakistan/DUHS24/2022 | EPI_ISL_14441435 |
| hCoV-19/Pakistan/DUHS25/2022 | EPI_ISL_14441436 |
| hCoV-19/Pakistan/DUHS26/2022 | EPI_ISL_14441437 |
| hCoV-19/Pakistan/DUHS28/2022 | EPI_ISL_14441438 |
| hCoV-19/Pakistan/DUHS30/2022 | EPI_ISL_14441439 |
| hCoV-19/Pakistan/DUHS32/2022 | EPI_ISL_14441440 |
| hCoV-19/Pakistan/DUHS-04/2022 | EPI_ISL_17445531 |
| hCoV-19/Pakistan/DUHS-06/2022 | EPI_ISL_17445533 |
| hCoV-19/Pakistan/DUHS-13/2022 | EPI_ISL_17445534 |
| hCoV-19/Pakistan/DUHS-15/2022 | EPI_ISL_17445535 |
| hCoV-19/Pakistan/DUHS-17/2022 | EPI_ISL_17445536 |
| hCoV-19/Pakistan/DUHS-18/2023 | EPI_ISL_17445537 |
| hCoV-19/Pakistan/DUHS-23/2023 | EPI_ISL_17445538 |
| hCoV-19/Pakistan/DUHS-02/2022 | EPI_ISL_17445918 |
| hCoV-19/Pakistan/DUHS-03/2022 | EPI_ISL_17445919 |
| hCoV-19/Pakistan/DUHS-07/2022 | EPI_ISL_17445920 |
| hCoV-19/Pakistan/DUHS-08/2022 | EPI_ISL_17445921 |
| hCoV-19/Pakistan/DUHS-09/2022 | EPI_ISL_17445922 |
| hCoV-19/Pakistan/DUHS-11/2022 | EPI_ISL_17445923 |
| hCoV-19/Pakistan/DUHS-12/2022 | EPI_ISL_17445924 |
| hCoV-19/Pakistan/DUHS-14/2022 | EPI_ISL_17445925 |
| hCoV-19/Pakistan/DUHS-16/2022 | EPI_ISL_17445926 |
| hCoV-19/Pakistan/DUHS-19/2023 | EPI_ISL_17445927 |
| hCoV-19/Pakistan/DUHS-20/2023 | EPI_ISL_17445928 |
| hCoV-19/Pakistan/DUHS-21/2023 | EPI_ISL_17445929 |
| hCoV-19/Pakistan/DUHS-22/2023 | EPI_ISL_17445930 |
| hCoV-19/Pakistan/BATCH-02-SAMPLE01/2023 | EPI_ISL_17474674 |
| hCoV-19/Pakistan/BATCH-02-SAMPLE02/2023 | EPI_ISL_17474675 |
| hCoV-19/Pakistan/BATCH-02-SAMPLE08/2023 | EPI_ISL_17474681 |
| hCoV-19/Pakistan/BATCH-02-SAMPLE16/2023 | EPI_ISL_17474689 |
| hCoV-19/Pakistan/BATCH-02-SAMPLE18/2023 | EPI_ISL_17474691 |
| hCoV-19/Pakistan/BATCH-02-SAMPLE19/2023 | EPI_ISL_17474692 |
| hCoV-19/Pakistan/DUHS-01/2024 | EPI_ISL_18788088 |
| hCoV-19/Pakistan/DUHS-03/2023 | EPI_ISL_18788090 |
| hCoV-19/Pakistan/DUHS-04/2023 | EPI_ISL_18788091 |
| hCoV-19/Pakistan/DUHS-05/2023 | EPI_ISL_18788092 |
| hCoV-19/Pakistan/DUHS-06/2023 | EPI_ISL_18788093 |
| hCoV-19/Pakistan/DUHS-07/2024 | EPI_ISL_18788094 |
| hCoV-19/Pakistan/DUHS-12/2024 | EPI_ISL_18831500 |
| hCoV-19/Pakistan/DUHS-20/2024 | EPI_ISL_18831508 |
| hCoV-19/Pakistan/DUHS-MN1/2021 | EPI_ISL_7861807 |
| hCoV-19/Pakistan/DUHS01/2021 | EPI_ISL_8317245 |
| hCoV-19/Pakistan/DUHS02/2021 | EPI_ISL_8317246 |
| hCoV-19/Pakistan/DUHS05/2021 | EPI_ISL_8317247 |
| hCoV-19/Pakistan/DUHS26/2021 | EPI_ISL_8486876 |
| hCoV-19/Pakistan/DUHS29/2021 | EPI_ISL_8487235 |
| hCoV-19/Pakistan/NIH-B135-S7/2021 | EPI_ISL_17814257 |
| hCoV-19/Pakistan/NIH-B135-S9/2021 | EPI_ISL_17814259 |
| hCoV-19/Pakistan/NIH-B142-S8/2021 | EPI_ISL_17973430 |
| hCoV-19/Pakistan/NIH-B152-S13/2021 | EPI_ISL_18075449 |
| hCoV-19/Pakistan/NIH-B152-S14/2021 | EPI_ISL_18075450 |
| hCoV-19/Pakistan/NIH-B154-S8/2021 | EPI_ISL_18123234 |
| hCoV-19/Pakistan/NIH-B154-S9/2021 | EPI_ISL_18123235 |
| hCoV-19/Pakistan/NIH-AHF-S24/2023 | EPI_ISL_18445462 |
| hCoV-19/Pakistan/UWARN-AKU-KHI-70395-S16/2024 | EPI_ISL_19192632 |
| hCoV-19/Pakistan/NIH-B169-S31/2024 | EPI_ISL_19221081 |
| hCoV-19/Saudi Arabia/KAUST489/2021 | EPI_ISL_16675001 |
| hCoV-19/Saudi Arabia/KFSHRC_20S/2021 | EPI_ISL_17276067 |
| hCoV-19/Saudi Arabia/KFSHRC_1O/2022 | EPI_ISL_17616339 |
| hCoV-19/Saudi Arabia/KFSHRC_30AJ/2023 | EPI_ISL_19640595 |
| hCoV-19/Saudi Arabia/KFSHRC_2AN/2024 | EPI_ISL_19642772 |
| hCoV-19/India/MP-ICMR-NIRTH-S13/2020 | EPI_ISL_19624138 |
| hCoV-19/India/DL-VRDL-AHRR-00062/2022 | EPI_ISL_19755601 |
| hCoV-19/India/DL-VRDL-AHRR-00079/2022 | EPI_ISL_19769580 |
| hCoV-19/India/GJ-INSACOG-GBRC-14511/2025 | EPI_ISL_20046871 |
| hCoV-19/India/MP-INSACOG-GBRC-14515/2025 | EPI_ISL_20046875 |
| hCoV-19/Henan/WIV-AY025/2020 | EPI_ISL_19765432 |
| hCoV-19/Henan/WIV-AY666/2020 | EPI_ISL_19765445 |
| hCoV-19/Hubei/WIV-YB0420_072/2020 | EPI_ISL_19765459 |
| hCoV-19/Shanxi/SXJCCDC-JCCX25066/2025 | EPI_ISL_19917607 |
| hCoV-19/Shanxi/SXJCCDC-JCCX25114/2025 | EPI_ISL_19917661 |
| hCoV-19/Bangladesh/ideSHi-031310/2021 | EPI_ISL_18875527 |
| hCoV-19/Bangladesh/ideSHi-2004226211/2020 | EPI_ISL_18875543 |
| hCoV-19/Bangladesh/icddrb-HCWC-02040158/2023 | EPI_ISL_19406608 |
| hCoV-19/Bangladesh/icddrb-HCWC-05020193/2024 | EPI_ISL_19406618 |
| hCoV-19/Bangladesh/icddr-b-1250503020/2025 | EPI_ISL_19894988 |
| hCoV-19/Australia/NSW-ICPMR-50518/2023 | EPI_ISL_18513410 |
| hCoV-19/Australia/NSW-ICPMR-50608/2023 | EPI_ISL_18513500 |
| hCoV-19/Australia/NSW-ICPMR-52522/2023 | EPI_ISL_18798760 |
| hCoV-19/Australia/VIC-VIDRL-00013149/2023 | EPI_ISL_18880531 |
| hCoV-19/Australia/QLD0x014175/2024 | EPI_ISL_19009767 |
| hCoV-19/Iran/NIC-30/2022 | EPI_ISL_16464027 |
| hCoV-19/Iran/NIC-34/2022 | EPI_ISL_16464031 |
| hCoV-19/Iran/NIC-9es-35/2023 | EPI_ISL_17324873 |
| hCoV-19/Iran/EF1016/2023 | EPI_ISL_18443026 |
| hCoV-19/Iran/NIC-25Mehr-4/2023 | EPI_ISL_19508829 |
| hCoV-19/USA/ND-NDDH-26187/2021 | EPI_ISL_19902720 |
| hCoV-19/USA/ND-NDDH-26198/2021 | EPI_ISL_19902731 |
| hCoV-19/USA/ND-NDDH-26216/2022 | EPI_ISL_19902776 |
| hCoV-19/USA/NY-SUNY-080/2025 | EPI_ISL_19906130 |
| hCoV-19/USA/NY-SUNY-081/2025 | EPI_ISL_19906131 |

This table provides the GISAID accession IDs of global and regional SARS-CoV-2 sequences included in the phylogenetic analysis. Sequences were selected based on lineage relevance, geographical representation (including India, China, Bangladesh, Iran, Saudi Arabia, USA, and Australia), and temporal overlap with samples collected during the study period. All sequences were downloaded from the GISAID EpiCoV database.
